# Factors associated with stunting in under-five children with environmental enteropathy in slum areas of Jimma town, Ethiopia

**DOI:** 10.3389/fnut.2024.1335961

**Published:** 2024-04-08

**Authors:** Rediet Regassa, Tefera Belachew, Markos Duguma, Dessalegn Tamiru

**Affiliations:** ^1^Department of Nutrition and Dietetics, Faculty of Public Health, Institute of Health, Jimma University, Jimma, Ethiopia; ^2^Jimma University Laboratory of Drug Quality (JuLaDQ) and School of Pharmacy, Jimma, Ethiopia

**Keywords:** stunting, environmental enteropathy, environmental enteric dysfunction, lactulose–mannitol test, WASH, malnutrition

## Abstract

**Introduction:**

Stunting is a major public health issue with a significant influence on the health and development of children in low-income countries, where it affects up to 32% of children. Nutritional intake is impacted by alterations in intestinal permeability and underlying chronic inflammation, which hinder children’s normal linear growth. Environmental enteropathy is a poorly understood condition with chronic intestinal inflammation. The purpose of this study was to identify the magnitude of stunting, change in growth, and factors associated with stunting and change in height for the age Z-score of children with an elevated lactulose-to-mannitol ratio.

**Methods:**

An observational follow-up study was conducted to follow children with an elevated lactulose-to-mannitol ratio for changes in their linear growth. A lactulose–mannitol test was performed to identify children with elevated lactulose-to-mannitol ratios, indicative of environmental enteropathy. After a 1-year follow-up, anthropometry was repeated to assess their linear growth. A multivariable logistic regression analysis was performed to identify the independent predictors for stunting in children with elevated lactulose-to-mannitol ratios. All tests were two-sided, and a *p*-value of <0.05 was considered significant.

**Results:**

The prevalence of stunting in children with an elevated L:M at baseline and end line was found to be 72.4% (95% CI: 60.3, 84.5) and 78.4% (95% CI: 66.7, 90.2), respectively. In a multivariate analysis, a low dietary diversity score (<4 food groups), presence of flies and insects in the toilet area, poor handwashing practices during a critical time, and MUAC z < −2 were significantly associated with stunting. Flies and insects in the toilet area and unsafe disposal of feces were significantly associated with changes in HAZ in children with elevated lactulose-to-mannitol ratios, an indicator of environmental enteropathy.

**Conclusion:**

Most of the children with an elevated lactulose-to-mannitol ratio in the study population were stunted, and no significant change in their linear growth was observed after 1-year follow-up. Therefore, further investigation and urgent intervention are needed to prevent environmental enteropathy and stunting among under-five children in this community who are exposed to very poor sanitary conditions and other risk factors for malnutrition.

## Introduction

Stunting affects one in five children before they reach the age of 5 years, making it a serious global public health concern. In low-income countries (LICs), where it is predicted to occur in 34.6% of cases, stunting is especially common (1). The prevalence decreased from 41.5% in 2000 to 30.7% in 2020 in Africa, even though the total number of cases increased from 54.4 million in 2000 to 61.4 million in 2020. Stunting in children is the most common problem worldwide with a height-for-age (HAZ) score of <−2. The World Health Organization (WHO) estimates that 155 million children worldwide, or 23% of children under the age of 5 years, are stunted (2).

In children, stunting is linked to irreversible harm and a host of detrimental health effects. It has been linked to children’s low developmental attainment and low IQ levels. Additionally, there is proof that children who are stunted are less likely to enroll in school, have a higher risk of mortality, and are more susceptible to infections (2).

Stunting is connected to both recurrent infections and inadequate nutrition. It can currently be caused by a variety of factors, including inadequate nutrition, poor hygiene, and recurrent infections. The complicated condition known as stunting can have many causes, with the most common being an imbalanced and inadequate diet and a lack of vitamins and other micronutrients. It also takes into account social elements, such as the makeup and resources of the family and the larger political and economic environments in which children live (3).

Studies from Africa, Asia, and South America have found increased lactulose-to-mannitol/L:M ratios [an indicator of environmental enteropathy (EE)] in asymptomatic children. A study of asymptomatic Gambian children aged 2–5 years found a correlation between L:M ratios and height for age (linear growth), which is hindered by underlying chronic inflammation and changes in intestinal architecture (4).

The biological level of stunting has been linked to three main hypotheses: EE, recurrent episodes of diarrhea, and infections (helminths) spread through the soil (5). Infants and young children are frequently exposed to fecal pathogens through the direct ingestion of fecally contaminated soil and/or animal feces, which is common in low-income environments. This exposure is linked to an increased risk of diarrhea, elevated markers of EED, and stunting (6).

In light of a study that revealed a significant knowledge gap in our understanding of growth stunting and its associated mortality that cannot be explained by food insecurity, the impact of subclinical yet pathological changes in the absorptive and immune functions of the gastrointestinal tract of infants and children in the developing world is being considered (7).

Stunting is caused by inadequate sanitation and hygiene, which results in EE, a subclinical condition that also causes diarrhea (8). EE is a vaguely defined condition of intestinal inflammation without obvious diarrhea that develops in people who are repeatedly exposed to substandard hygiene and sanitation. Pathologically, it is marked by inflammation and villous blunting in the small intestine. It is linked to stunting, poor cognitive development, and oral vaccine failure in children from low-income nations (9, 10). A solution to EE is urgently needed because of the burden of malnutrition alone, which affects 25% of children worldwide and is estimated to cause over a million deaths annually due to increased susceptibility to infection (9, 10).

Stunting continues to be a significant public health burden despite the Ethiopian government’s adoption and implementation of the national nutrition programs, strategies, and infant and young child feeding intervention programs in recent years to address this issue (11).

Numerous studies show that malnutrition is highly prevalent in underdeveloped nations and suggest nutritional interventions such as balanced diets, breastfeeding, and micronutrient supplements; yet, there is no discernible difference in the rate of malnutrition reduction. This indicates that there is a gap in the solution to decreasing malnutrition. As infection is one of the causes of malnutrition and also studies were done on the association of infection and malnutrition with the direct effect of clinical manifestation diarrhea, there is little evidence of an indirect effect with subclinical condition (environmental enteropathy). Therefore, this study aimed to observe the change in growth of children with an elevated lactulose to mannitol ratio/Indicative of Environmental Enteropathy.

## Materials and methods

### Study design

An observational follow-up study was used to determine if a single L: M ratio predicts subsequent linear growth in slum areas of Jimma town among children aged 12–59 months with an elevated lactulose-to-mannitol ratio. Jimma town is located in the southwest part of Ethiopia, at a distance of 357 km from the capital city, Addis Ababa. The town has 13 urban kebeles and 4 rural kebeles, with a total population of 207,573. The temperature in the area typically varies from 48°F to 83°F throughout the year and is rarely below 42°F or above 88°F. The wet season is mostly cloudy, while the dry season is partly cloudy. The town is bounded by Kersa woreda in the east, Manna woreda in the west, Mana and Seka woreda in the north, and Seka woreda in the south.

### Data collection procedure

Native Oromia speakers who worked as lab technicians and health extension workers were hired from health posts and centers to collect data, and they received 2 days of training before beginning their work. Socioeconomic and demographic details, child feeding habits, personal hygiene routines, food preparation techniques, housing conditions, and morbidity reports were included in the data collection tool. Using an observation checklist, data collectors recorded observations of the water storage practice, restroom, and handwashing station. The repeated 24-h dietary recall method was used to calculate the dietary diversity score (DDS). The children’s caretakers were asked to recall everything their children had eaten or consumed during the preceding 24 h. The total number of distinct food groups that the children consumed in the 24 h before the assessment was added up to determine the DDS (5).

### Measurements

The appropriate tools were utilized to gather data and apply accepted measurement practices. Children under the age of 2 years should lie on a suitable board to have their length measured, and children over the age of 2 years should stand up to have their height measured. For instance, the children were weighed in their underwear without shoes, and their mid-upper circumference (MUAC), weight, and height were measured appropriately. Children who met the WHO 2006 guidelines, the U.S. National Center for Health Statistics (NCHS), the Centers for Disease Control and Prevention, and the WHO reference population were classified as stunted, underweight, wasted, and thin, respectively.

#### Weight

The research subjects were weighed while barefoot on a hard board covering a level surface, either in a diaper or light underwear. A beam balance was utilized for this purpose, which was routinely calibrated against known weights. The weight was measured to the nearest 100 g.

#### Height

For children under the age of 2 years, the standard measuring board (sliding board) was used to measure height; for children over the age of 2 years, a stadiometer—a height-measuring instrument with a precision of 0.1 cm—was used to measure height in a standing position.

#### MUAC

Using a non-stretchable tap, the left arm’s MUAC was measured halfway between the olecranon and acromion processes, to the nearest 1 mm.

### Urine collection

Two weeks before the lactulose/mannitol solution was administered, 300 children (140 male toddlers and 160 female toddlers) in the age range of 12–59 months (median age of 3 years) who were at risk of developing EE and had no significant medical histories or gastrointestinal symptoms were included in the test at baseline. The subjects drank the test sugar solution, which contained 250 mg/mL of lactulose and 50 mg/mL of mannitol, at a dose of 2 mL/kg up to a maximum of 20 mL, following an overnight fast. Urine flow was allowed to increase with a liberal intake of water after 30 min. Food was permitted after the initial 3 h.

Before the children consumed the test sugar solution, they were asked to empty their bladders. A urine collection bag (McKesson Medical-Surgical, Inc., MFR #4822, and Richmond, VA) was placed and changed as needed during the 5-h duration (9). After voiding, samples were aliquoted and kept on ice to prevent microbial growth. One or two drops of chlorhexidine (20 mg/mL) were added to measure the volume of urine. Before testing, urine aliquots were kept at −80°C (12). After the study was finished, the samples were kept in the Jimma University laboratory at −80°C until they were shipped on dry ice to the Ethiopian Conformity Assessment Enterprise laboratory for analysis.

### Sample size determination

A total of 300 children in the age group of 12–59 months were selected by using the single population proportion formula with an assumption of an estimated prevalence (p) of EE of 50%, a 5% margin of error (d), and a 95% confidence level. A simple random sampling technique was used to select the study subjects. Of the 300 children who were eligible for the test, 58 were found to have an increased lactulose–mannitol ratio, an indicator of EE. Fifty-eight children (58) with elevated lactulose-to-mannitol ratios were followed for change in linear growth. After a 1-year follow-up, seven children were lost. As a result, data collection and analysis were performed on 51 children with an elevated lactulose-to-mannitol ratio, an indicator of EE.

### Laboratory procedure

Sample preparation: A measure of 0.5 g of washed cation exchange resin was added to 2 mL of the thawed urine specimen, vortexed for 10 s and centrifuged for 10 min at 3000 rpm. The supernatant layer was withdrawn and filtered through a 0.2 L filter to inject into the high performance liquid chromatography (HPLC) system for analysis (13).

#### Chromatographic conditions and the HPLC system

The samples were analyzed using an Agilent 1260 Infinity Series HPLC system (Model SP 8810; Spectra-Physics, San Jose, CA) along with a carbohydrate column (4.6 × 150 mm, 5 μm; Zorbax, United States). The mobile phase was a mixture of acetonitrile/HPLC-grade water (70/30%v/v). The HPLC analysis was conducted at the column temperature, injection volume, and flow rate of ambient temperature, 10.0 μL and 1.0 mL/min, respectively. Calibration curves were prepared by analyzing appropriate concentrations of each compound in distilled water and plotting the peak height obtained at 1.0 × l0 refractive index unit (full scale) sensitivity of the detector. Urinary concentrations of sugar probes were calculated from the calibration curves by peak-area analysis (12) (Supplementary Figures S1, S2). Except for the lactulose–mannitol test, the baseline procedure was repeated after the follow-up period.

### Variables of the study

The study was conducted to identify the outcome variable of stunting in children who had an elevated lactulose-to-mannitol ratio, an indicator of EE. Indicators for water, sanitation, and hygiene (WASH) problems and the DDS were taken as predictors for the outcome variable.

### Data source

Face-to-face interviews with caregivers of children under the age of 5 years were conducted to gather information on sociodemographic characteristics, health status, and water, sanitation, and hygiene practices. The questionnaires were structured. To measure intestinal permeability, mannitol and lactulose solutions were administered, and then a urine sample was taken. Using an observation checklist, data collectors recorded observations of the water storage practice, restroom, and handwashing station.

### Data analysis

The lactulose–mannitol test was performed on 300 children who were eligible for the test. In the test, mannitol recovery rates indicated absorptive capacity; lactulose recovery rates indicated permeability; and higher L:M ratios indicated greater intestinal abnormality, or EED. In this study, the lactulose–mannitol ratio, L: M, was calculated as the ratio of the lactulose-to-mannitol concentrations in the urine.

Data were entered using EpiData version 3.1 and were subsequently imported into SPSS version 26 for analysis. The study variables were compiled using descriptive statistics such as cross-tabulation and frequency distribution. The crude and adjusted odds ratios with 95% confidence intervals were estimated using both univariate and multivariable logistic regression methods. To account for possible confounding, a backward step-wise variable selection method was used. Only variables that achieved *p*-values of ≤0.25 in the bivariate analysis were included in the multivariable logistic regression analyses to control for associations among the independent variables, thereby eliminating excess variables and unstable estimates in the final model. The Hosmer–Lemeshow test was used to test the goodness of fit of the model, and a *p*-value of >0.05 was considered to be fit. When a regression coefficient was set at less than 2.0, the standard errors indicated that there was no multicollinearity among the independent variables. Using WHO Anthro software, the children’s height, length, and weight were converted to MUAC, the Z-score of height for age, and the weight for height and age. A standard deviation of less than 2 was considered stunted, wasted, underweight, and thin for HAZ, WHZ, WAZ, and MUACZ, respectively. The change in HAZ was determined by the difference between the end line and baseline height for age z-score. The difference of <0.25 Z was taken as a loss, and > 0.25 Z was taken as a gain in height for age z-score (14).

### Ethical consideration

The University of Jimma Ethical Review Board, the College of Medicine and Health Sciences, the Institute of Public Health, and the Oromia Regional Health Bureau’s Ethics Committee all granted their ethical approval with the reference no of IHRPGD/456/2020. Official consent was obtained from the administrative and health offices in each study district before the start of the study. The participants received an explanation of the study’s goals and significance. As per the Declaration of Helsinki, a parent or guardian’s written informed consent was obtained. The study participants’ confidentiality was maintained, and participant identification by name was avoided. A nearby medical facility was contacted for follow-up and management of all the wasted and stunted children with an elevated lactulose-to-mannitol ratio (>0.15).

## Results

The mean SD % lactulose excreted was found to be 0.6 ± 0.1, and the mean SD % mannitol excreted was found to be 4.5 ± 1.6. The mean SD of the L:M ratio was found to be 0.27± 0.06. The lactulose-to-mannitol ratio of >0.15 was taken in children with EE. Of the study participants, the lactulose-to-mannitol ratio of 58 children was >0.15/with EED, while those of 242 were < 0.15/ without EED (Table 1).

**Table 1:** Lactulose–mannitol test to determine the magnitude of environmental enteropathy (*n* = 300).

% lactulose excreted (mean SD)	% mannitol excreted (mean SD)	% L:M excreted (mean SD)	% L:M<0.15/No EED		L: M > 0.15/EED	
			Number	Percent	Number	Percent
0.6 ± 0.1	4.5 ± 1.6	0.27 ± 0.06	242	80.7	58	19.3

EED, environmental enteric dysfunction; SD, standard deviation; L:M, lactulose-to-mannitol ratio.

The mean age (±SD) of study participants was 1.2 (+0.46). The majority of the children (i.e.,) 33(64.7%) of them, with an elevated lactulose-to-mannitol ratio fall within the age group of 1–3 years. Most of the children, 28 (54.9%), were male toddlers, and 44(86.3%) of the caregivers were between 18 and 35 years of age. The majority of children, 34(66.7%), with elevated lactulose-to-mannitol ratios were from families with a size of ≥5, and 36 (70.6%) of the caregivers were with an income of <3,000 Birr (Table 2).

**Table 2:** Socioeconomic and demographic characteristics of children with an elevated lactulose-to-mannitol ratio/environmental enteropathy and their caregivers at end line and follow-up (*n* = 51).

Variables	Category	Number	Percent
Child age	1–3	33	64.7
	4–5	18	35.3
			
Child sex	Male	28	54.9
	Female	23	45.1
Age of care givers	18–35	44	86.3
	>35 years old	7	13.7
Household family size	≥5	34	66.7
	<5	17	33.3
Marital status	Married	33	64.7
	Divorced	14	27.5
	Widowed	4	7.8
Paternal education	No education	10	19.6
	Primary	18	35.3
	Secondary	23	45.1
Maternal occupation	Self/Government Employed	11	21.5
	Unemployed	40	78.5
Parental occupation	Self/Government Employed	16	31.4
	Unemployed	35	68.6
Monthly income (ETB)	<3,000 ETB	36	70.6
	≥ 3,000 ETB	15	29.4

**Figure 1:**
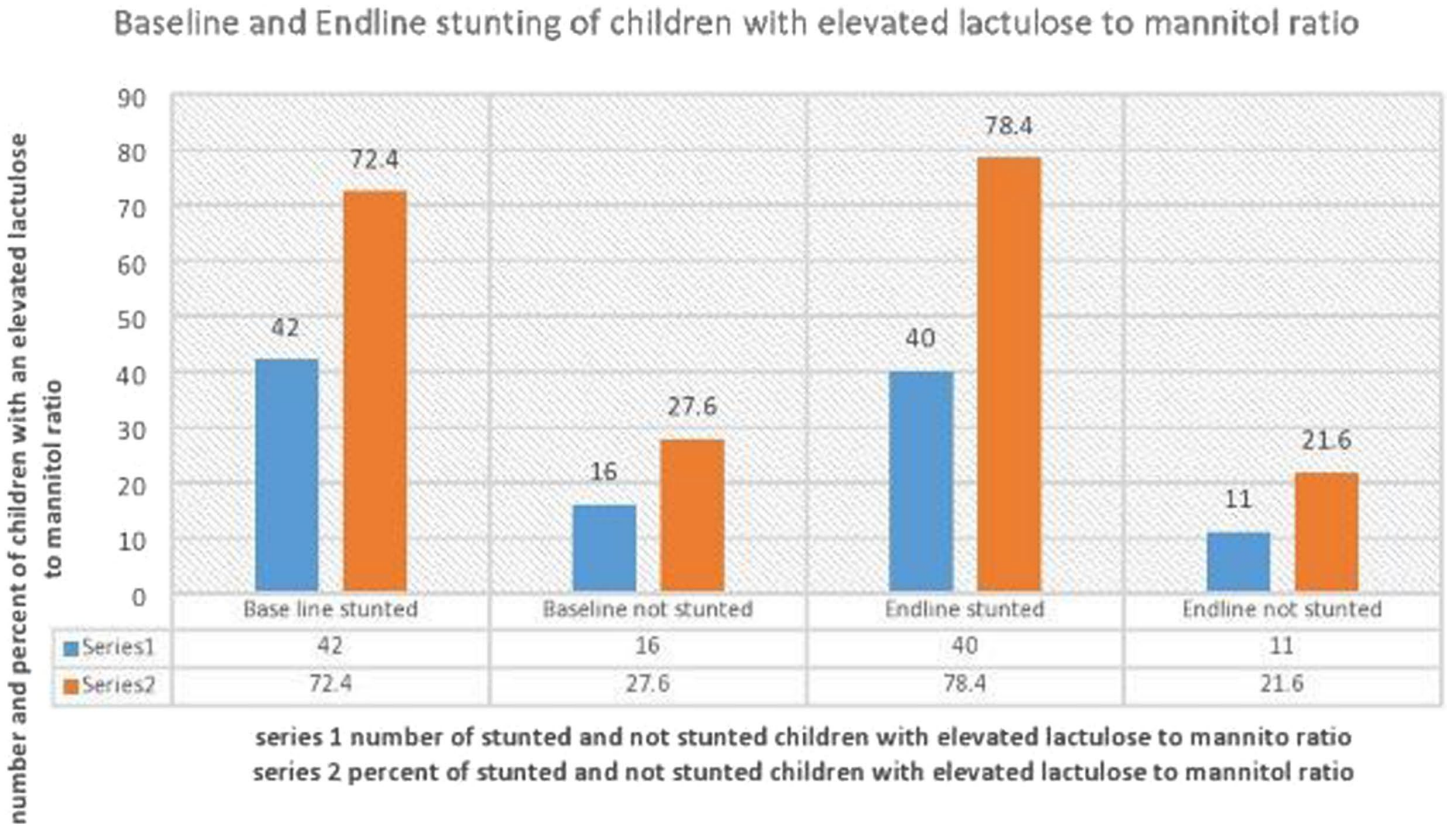
Distribution of stunting at baseline and end line in children with elevated lactulose to mannitol ratio/indicative of Environmental Enteropathy.

The magnitude of stunting in children with an elevated L:M at baseline and end line was 42 (72.4, 95%CI: 60.3, 84.5) and 40 (78.4, 95%CI: 66.7, 90.2), respectively. The average HAZ of children at baseline and end line was found to be −1.84 z-score and − 2.46 z-score, respectively, with a difference of −0.6 z-score (Figure 1).

At the end line, most of the children with elevated lactulose-to-mannitol ratios from the age groups of 1–3 years,27(81.8%), from households with DDS of <4, 31 (88.6%), from practicing less than three critical times of handwashing, 25 (80.6%), from unsafe solid waste disposal, 25 (78.1%), from households with unsafe disposal of feces, 28 (80%), from being exposed to flies and insects at the toilet area, 23 (88.5%), and who are thinned, 28 (75.7%) were more stunted than their references.

Concerning the change in height for age at baseline and end line during the follow-up period, the majority of the children from age groups of 1–3 years, 25 (75.8%), from households with DDS of <4, 27 (77.1%), from households with less than three critical times of handwashing, 22 (71%), from households with unsafe disposal of feces, 28 (80%), from being exposed to flies and insects at the toilet area, 30 (81.1%), and who are thinned, 26 (70.3%), were more likely to lose their height for age z than their references. In the bivariate analysis, at the end line, children from the age groups of 1–3 years (COR = 1.578; 95% CI: 0.148–2.248, *p* = 0.429), those practicing less than three critical times of handwashing (COR = 1.389; 95% CI: 0.361–5.349, COR = 1.389; 95%CI:0.361–5.349, *p* = 0.633), those from households with unsafe disposal of feces (COR = 1.333, 95%CI: 0.328–5.419, *p* = 0.688), and children who are thinned (COR = 1.929, 95%CI: 0.361–10.294, *p* = 0.442) were more likely to be stunted than their references. Children with a DDS of <4 (*p* = 0.014) and who were exposed to flies and insects in the toilet area (*p* = 0.004) were significantly associated with stunting.

Children from the age groups of 1–3 years (COR = 1.640, 95%CI: 0.181–2.262, *p* = 0.488) and children who are thinned (COR = 1.551, 95%CI: 0.361–6.669, *p* = 0.555) were more likely to lose their height for the age Z-score than their references. Children who were exposed to flies and insects in the toilet area (*p* = 0.032) and unsafe disposal of feces (*p* = 0.044) were significantly associated with the loss of their height for the age Z-score during the follow-up period (Table 3).

**Table 3:** Bivariate analysis of factors associated with end line and change in HAZ of children with an elevated lactulose-to-mannitol ratio (*n* = 51).

Variables	End line stunted						Change in HAZ					
	Yes		No		*p*-value	(COR,95% CI)	Lose <0.25		Gain >0.25		*p*-value	**(COR, 95% CI)**
	No	%	No	%			No	%	No	%		
Age												
1–3 years	27	81.8	6	18.2	0.429	1.578(0.148–2.248)	25	75.8	8	24.2	0.488	1.640(0.181–2.262)
4–5 years	13	72.2	5	27.8			12	66.7	6	33.3		
DDS <4 food	31	88.6	4	11.4	0.014	0.166(0.039–0.697)	27	77.1	8	22.9	0.281	0.494(0.137–1.782)
Groups >4 food groups	9	56.2	7	43.8			10	62.5	6	37.5		
Solid waste disposal												
Unsafe	25	78.1	7	21.9	0.945	0.952(0.238–3.806)	14	73.7	5	26.3	0.889	0.913(0.254–3.28)
Safe	15	78.9	4	21.1			23	71.9	9	28.1		
Practice less than 3 times of critical hand washing												
Yes	25	80.6	6	19.4	0.633	1.389(0.361–5.349)	22	71	9	29	0.753	0.815(0.228–2.916)
No	15	75	5	25			15	75	5	25		
Flies and insects at the toilet area												
Yes	23	88.5	3	11.5	0.004	0.277(0.064–1.203)	30	81.1	7	18.9	0.032	0.233(0.062-–0.884)
No	17	68	8	32			3	50	7	50		
Disposal of feces												
Unsafe	28	80	7	20	0.688	1.333(0.328–5.419)	28	80	7	20	0.044	3.111(0.857–11.291)
Safe	12	75	4	25			9	56.2	7	43.8		
Muac Z												
Thinned	28	75.7	9	24.3	0.442	1.929(0.361–10.294)	26	70.3	11	29.7	0.555	1.551(0.361–6.669)
Not thinned	12	85.7	2	14.3			11	78.6	3	21.4		

COR, crude odds ratio. Change in HAZ is the difference between the endline and baseline height for age Z-score HAZ gain is a difference in the endline and baseline height for age of > 0.25 Z-score. HAZ loss is a difference in the endline and baseline height for age of < 0.25 Z-score. Statistically significant at a *p*-value of < 0.05.

To rule out excess variables and unstable estimates in the final model, only variables that reached a *p*-value of less than 0.25 in the bivariate analysis were included in the multivariable analysis. After adjustment for potential confounders, at the end line, children from DDS <4 food groups, from unsafe solid waste disposal, from practicing handwashing less than three critical times of hand washing, and who are thinned were significantly associated with stunting of children with elevated lactulose-to-mannitol ratios.

Children who were exposed to flies and insects in the toilet area and unsafe disposal of feces were significantly associated with the loss of height for the age Z-score during the follow-up period. Children from the age groups of 1–3 years and MUAC z < −2 were more likely to lose their height for the age Z-score than their references (Table 4).

**Table 4:** Multivariate analysis for factors associated with end-line stunting and change in height for age z-score of children with elevated lactulose-to-mannitol ratio at end line and follow-up (*n* = 51).

		AOR(95%CI)	*p*-value	AOR(95%CI)	*p*-value
Age	1–3	0.148(0.018–2.082)	0.189	0.475(0.089–2.527)	0.383
	4–5	1			
DDS	Not diversified	1.227(0.233–6.457)	0.009	2.534(0.163–39,299)	0.506
	Diversified	1			
Flies and insects at the toilet area	Yes	0.505(0.112–2.274)	0.025	0.004(0.003–1.201)	0.045
	No	1			
Solid waste disposal	Unsafe	1.242(0.255–6.050)	0.789	1.136(0.244–5.281)	0.871
	Safe	1			
Practice less than three critical times of hand washing	Yes	2.768 (0.342–10.234)	0.034	0.898(0.160–5.040)	0.903
	No	1			
Disposal of feces	Unsafe	2.174(0.397–11.907)	0.371	5.015(0.902–27,871)	0.035
	Safe	1			
MUAC z	Thinned	0.111(0.014–1.906)	0.040	1.652(0.284–9.595)	0.576
	Not thinned	1			

AOR, Adjusted odds ratio. Statistically significant at a *p*-value of < 0.05.

## Discussion

For as long as childhood EED has been acknowledged as a condition, it has been linked to stunting or low height for age. However, which of these two factors is the main cause and which is the result is not evident (5). This study used the lactulose–mannitol test to identify children with an increased lactulose-to-mannitol ratio, an indicator of EE. The lactulose–mannitol test is widely used to assess intestinal absorptive capacity and permeability. Among studies reporting LM ratios (or separate mannitol and lactulose excretion values) and growth in children, most, but not all, found inverse associations with linear growth (13). This study aimed to indicate the magnitude of stunting, change in growth, and factors associated with stunting in children with an elevated lactulose-to-mannitol ratio during the follow-up period. Severe, irreversible physical, physiological, and cognitive damage brought on by chronic malnutrition in early life is manifested as stunting (15). Stunted children living in unhygienic settings have a high prevalence of environmental enteric dysfunction, or EED, which is widespread in impoverished nations with few resources (2).

According to the findings of this study, the magnitude of stunting in children with an elevated lactulose-to-mannitol ratio was high at baseline and end line, with a little difference in change in height for the age Z-score between baseline and end line (the loss in height for the age Z-score was much greater than the gain). This is similar to different studies that support the idea that children with a high lactulose-to-mannitol ratio, indicative of EE, are at risk of malnutrition (9, 10).

Concerning factors associated with end-line stunting and the change between baseline and end-line height for the age Z-score, in this study, there is no significant difference between baseline and end-line height for age Z (much less loss than gain in HAZ). As a result, factors that determine end-line stunting were also factors for change in HAZ. In multivariate analysis, children from households of DDS <4 food groups, from households of practicing hand washing less than three critical times of hand washing, from being exposed to toilets with flies and insects, and with MUAC z < −2 were significantly associated with stunting of children with elevated lactulose-to-mannitol ratios. According to the FAO guidelines, the minimum DDS from the seven food groups on a scale of 0–7 was found in at least four of the seven groups: (1) staples (cereals, grains, roots, and tubers); (2) dairy products; (3) animal or flesh foods; (4) legumes and nuts; (5) vitamin A-rich fruits and vegetables; (6) eggs; and (7) other fruits and vegetables (16). Children with lower DDSs are more susceptible to infection. As one of the causes of stunting is low DDS, children with low DDSs were more susceptible to infection. The findings of this study indicated that children from households with a DDS of <4 had a significant association with an elevated lactulose-to-mannitol ratio and, in turn, were stunted. They are also more likely to lose their height for the age Z-score. This is similar to different studies (17, 18) that indicated that low DDS, infection, and stunting are in a vicious cycle. Young children who live in underdeveloped areas without access to clean water or proper sanitation are more likely to experience recurring and multiple intestinal infections, both with and without overt diarrheal illnesses (4). There is a cyclical association between vulnerability to infection and inadequate nutrition. A diet low in nutrients increases the risk of contracting infectious diseases, which in turn causes immunological dysfunction and metabolic reactions that further modify nutritional status and, when feasible, associated physiological mechanisms (17). The F Diagram’s “fluids, fingers, fields, flies, and food” channel, or the fecal–oral route, is believed to be a key conduit for enteric infections (18).

A high lactulose-to-mannitol ratio (EED) is linked to problems with WASH (9, 10). Due to the problem with WASH, the majority of children are more likely to become infected, which leads to EED (9, 10), which can also run the danger of becoming stunted (19).

This study was conducted in a slum with poor sanitation, hygiene, and access to water. Studies have shown that, while there are many potential causes of EE, inadequate sanitation, hygiene, and water quality are associated with a higher incidence of the condition. Environmental enteric dysfunction has also been linked to unsafe water, poor sanitation, and poor hygiene practices (9, 10).

Due to numerous variables, including overcrowding, poor sanitation, and inadequate drinking water supply in residential areas, which are linked to lower socioeconomic levels, children living in urban slums are extremely vulnerable to stunting and nutritional failure (20).

The health and nutritional status of children living in impoverished areas are significantly threatened by factors such as inadequate sanitation, crowded living situations, low drinking water quality, and substandard housing (21).

Furthermore, slum dwellers frequently use shared restrooms due to inadequate waste disposal facilities, and some even utilize open spaces or plastic bags, also known as “flying toilets,” to relieve themselves. When crawling or playing in contaminated areas linked to improper waste disposal techniques, children may ingest contaminated substances such as pica and fomites via fingers (21). As a result, children living in such conditions are always at a high risk of undernutrition and other health problems.

Stunting has been linked to poor WASH, repeated episodes of diarrhea, helminth infections in the soil, and EE (10). Children between the ages of 6 and 36 months exhibited an increase in height when WASH interventions, including sanitation education, hand washing with soap, the availability of sanitary facilities at HHs, a clean atmosphere at HHs, and separate housing for animals, were implemented (10). Similarly, the intervention has the objective of preventing the ingestion of harmful microbes by interrupting fecal–oral transmission (22).

The prevalence of childhood malnutrition and stunting can be decreased by providing access to clean drinking water, sanitation, and hygiene (WASH). These factors can prevent diarrhea, parasitic infections, and EE, which can impair the body’s capacity to absorb nutrients and impede healthy growth and cognitive development (11).

Stunting is a possibility for children with high lactulose–mannitol ratios (an indicator of EE). Numerous studies have revealed a connection between EE and stunting in children (9, 10). Globally, environmental enteric dysfunction, or EED, is the most common disorder linked to poor gut health. It is common in children from rural Africa and is linked to stunting (7).

According to this study, children who are thinned are significantly associated with stunting in children with an elevated lactulose-to-mannitol ratio. This is also similar to the study that indicated the effect of intestinal permeability on the absorption of nutrients, which leads to being wasted, thinned, and stunted (17). A follow-up study conducted on Kenyan children also indicated that a change in MUAC has a strong correlation with a change in HAZ (14). The possible justification for this might be that most of the children with an elevated lactulose-to-mannitol ratio, indicative of EE, were thinner than their references.

The WHO has designated stunting as a global health priority, and by 2025, it wants to see a 40% decrease in the number of children who are stunted (23). Accordingly, the capacity to identify, effectively prevent, and treat EED is severely constrained by significant knowledge gaps on the disease’s pathophysiology and relationship to stunting (19). Therefore, the goal of this study was to pinpoint EED as one of the elements causing stunting in children under the age of 5 years in the study area.

### Limitation

The lactulose–mannitol test was not conducted throughout the follow-up period because there were not enough facilities in the nation where this study was conducted to diagnose intestinal permeability or EE. Owing to a lack of resources, further factors that affect children’s linear growth are not included here.

## Conclusion

The majority of children with higher lactulose-to-mannitol ratios were stunted. As the study was conducted in impoverished locations and there is a cyclical relationship between intestinal leakage/malabsorption, infection, and stunting, there was no significant change in the growth of children with a high lactulose-to-mannitol ratio (EE) during the follow-up period. Based on the study’s findings, we suggest an intervention strategy that would target the following responsible sectors: the health sector to increase public awareness of ways to prevent problems associated with inadequate WASH and the WASH sector to concentrate on areas lacking WASH facilities. Additionally, efforts would focus on EE diagnostic measures for children to pinpoint the precise cause of stunting and reduce its prevalence both nationally and in the study area.

## Data availability statement

The datasets presented in this article are not readily available because based on the policy of the Institutional Review Board of Jimma University, data for the Ph.D. study is restricted until the candidate finishes his plan. Requests to access the datasets should be directed to IRB (institutional review board).

## Ethics statement

The studies involving humans were approved by the Institutional Review Board, Institute of Health, Jimma University, e-mail: ethicsjuirb@gmail.com. The studies were conducted in accordance with the local legislation and institutional requirements. Written informed consent for participation in this study was provided by the participants’ legal guardians/next of kin.

## Author contributions

RR: Conceptualization, Data curation, Formal analysis, Funding acquisition, Investigation, Methodology, Resources, Software, Supervision, Validation, Visualization, Writing – original draft, Writing – review & editing. TB: Conceptualization, Data curation, Formal analysis, Investigation, Methodology, Resources, Software, Supervision, Validation, Visualization, Writing – original draft, Writing – review & editing. MD: Conceptualization, Data curation, Formal analysis, Investigation, Methodology, Resources, Software, Supervision, Validation, Visualization, Writing – original draft, Writing – review & editing. DT: Conceptualization, Data curation, Formal analysis, Investigation, Methodology, Resources, Software, Supervision, Validation, Visualization, Writing – original draft, Writing – review & editing.
