# Supplementary figures and images for: Factors associated with stunting in under-five children with environmental enteropathy in slum areas of Jimma town, Ethiopia
Source: Front Nutr. 2024 Apr 8;11:1335961. doi: 10.3389/fnut.2024.1335961 (PMC11034484; doi:10.3389/fnut.2024.1335961)

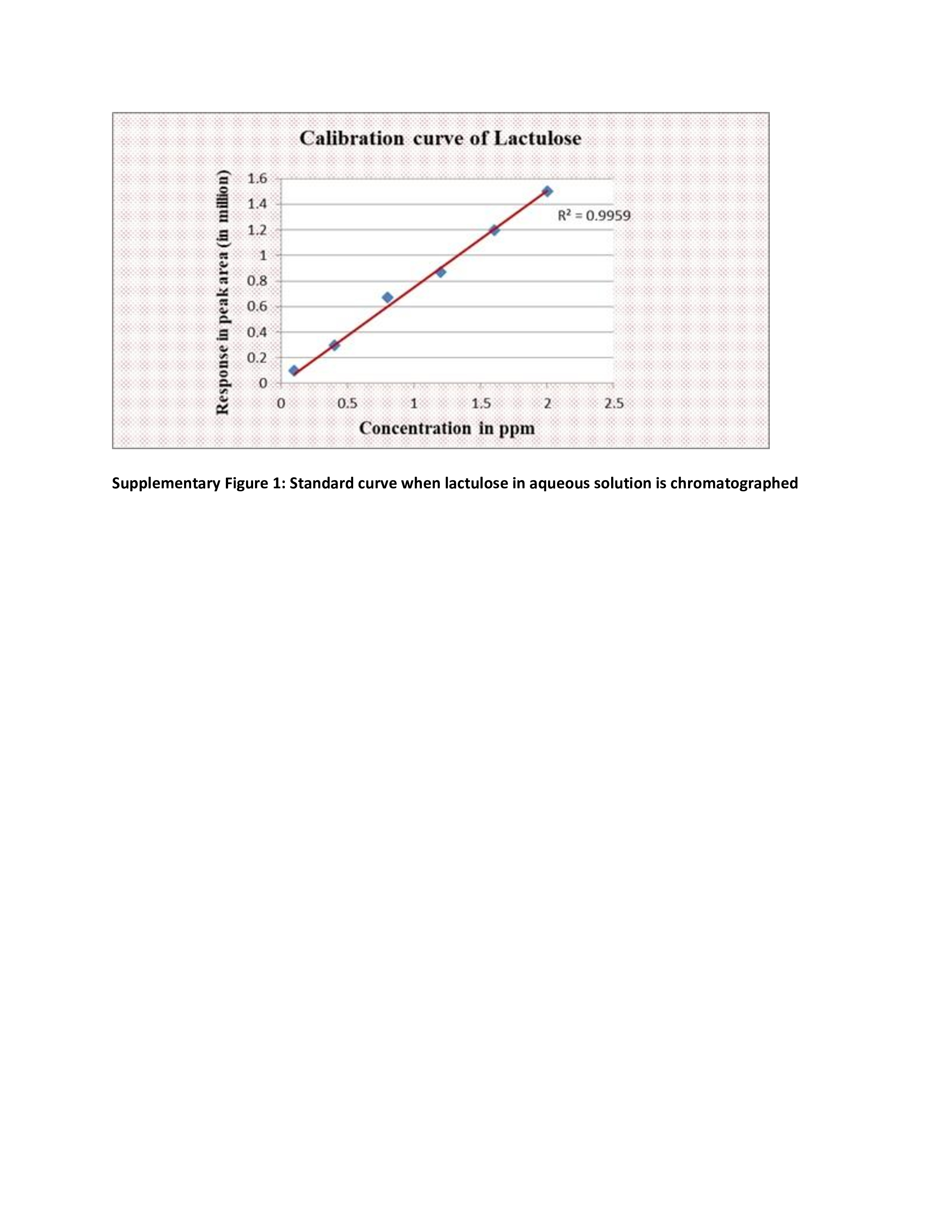

Supplement: Supplementary file 1 [file Image_1.jpg]

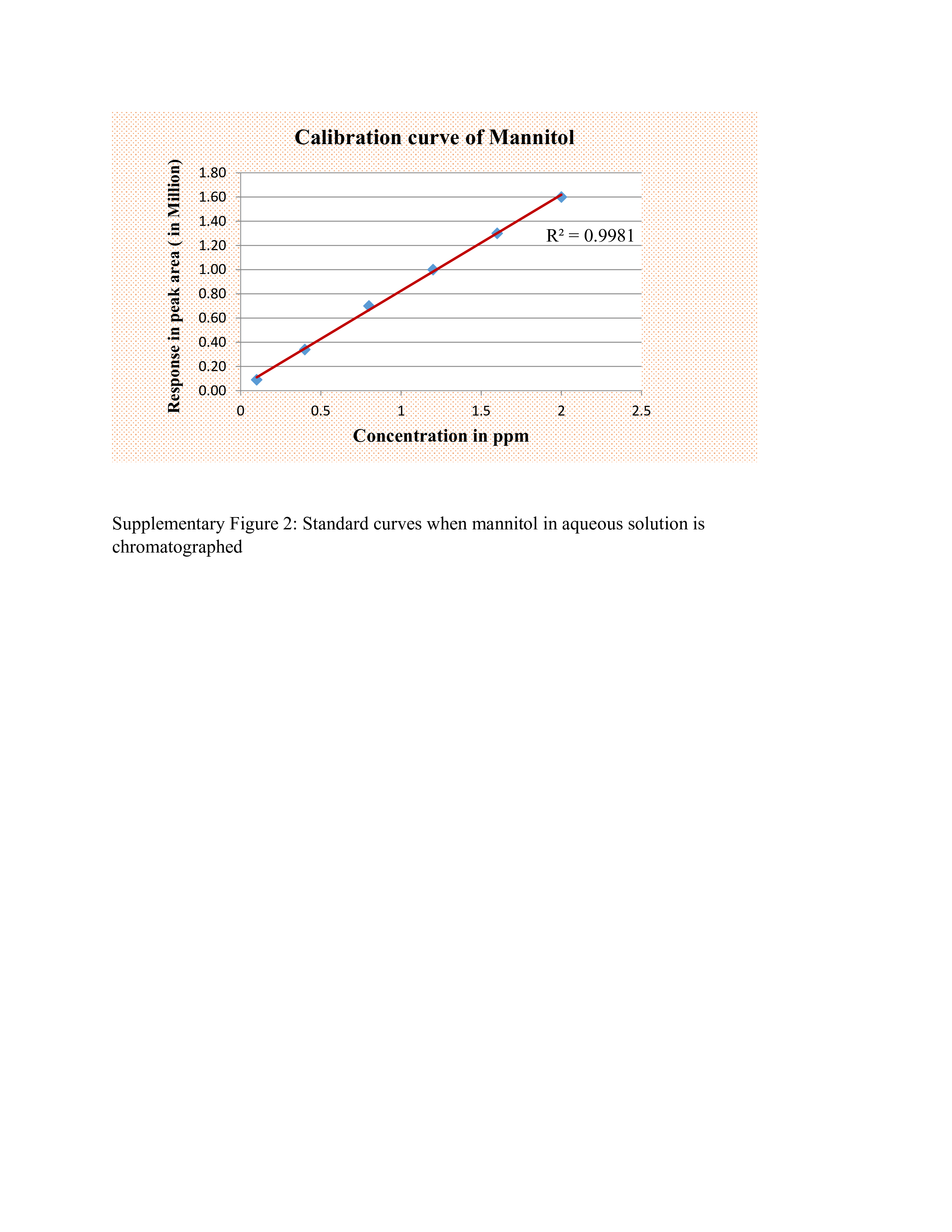

Supplement: Supplementary file 2 [file Image_2.jpg]
